# Integrative multi-omics analysis identifies *CCDC69* as a marker associated with germinal center B-cell infiltration in TNBC: development and validation

**DOI:** 10.1590/1414-431X2026e15616

**Published:** 2026-10-12

**Authors:** Jinjiao Li

**Affiliations:** 1 Department of Breast, Thyroid and Burn Surgery, The People's Hospital of Wenshan Prefecture, Wenshan, China

**Keywords:** CCDC69, Triple-negative breast cancer, Germinal center B cells, Tumor immune microenvironment, Spatial transcriptomics

## Abstract

Although coiled-coil domain containing 69 (*CCDC69*) has been implicated in tumor biology, its role in triple-negative breast cancer (TNBC) and immune regulation remains unclear. Bulk transcriptomic data, single-cell RNA sequencing datasets, and spatial transcriptomics were used to investigate the role of *CCDC69* in TNBC. Germinal center B-cell (GCB) signatures were quantified using single-sample gene set enrichment analysis (ssGSEA), and their associations with *CCDC69* expression were assessed using correlation, ROC, and logistic regression analyses. An independent external TNBC cohort (GSE58812) was used to validate the association between *CCDC69* expression, GCB infiltration, and survival outcomes. *CCDC69* expression was significantly downregulated in breast cancer tissues but was associated with favorable survival outcomes in TNBC patients. *CCDC69* expression positively correlated with multiple immune cell populations, particularly B cells and plasma cells. Single-cell RNA sequencing demonstrated that *CCDC69* expression was enriched in B-lineage cells, especially GCB. Spatial transcriptomic analyses further showed that *CCDC69*-high regions were spatially co-localized with B-cell-rich and germinal center-like immune niches. In the TCGA TNBC cohort, *CCDC69* expression was strongly associated with GCB infiltration and effectively distinguished GCB-high tumors. Logistic regression analysis confirmed that *CCDC69* was independently associated with GCB enrichment. Findings were validated in the GSE58812 cohort. *CCDC69* expression correlated with GCB-related signatures, showed good discriminative performance in identifying GCB-high tumors, and was associated with improved patient outcomes when combined with GCB enrichment. *CCDC69* is closely associated with B-cell-mediated immune responses and germinal center niches in TNBC. Our findings suggest that *CCDC69* may serve as a robust biomarker of immune activation and favorable prognosis.

## Introduction

Triple-negative breast cancer (TNBC) is an aggressive molecular subtype of breast cancer characterized by the absence of estrogen receptor, progesterone receptor, and HER2 expression (1). Compared with other breast cancer subtypes, TNBC exhibits higher rates of recurrence and metastasis and lacks effective targeted therapies, resulting in poor clinical outcomes. In recent years, increasing evidence has highlighted the crucial role of the tumor immune microenvironment in TNBC progression and therapeutic response (2). In particular, immune-rich TNBC tumors are often associated with improved prognosis and better responses to immunotherapy (3-5).

Among immune components within the tumor microenvironment, B cells have emerged as important regulators of antitumor immunity (6). Tumor-infiltrating B cells contribute to immune responses through antigen presentation, antibody production, and interactions with T cells (6,7). Notably, B cells organized in tertiary lymphoid structures (TLS) often form germinal center-like regions that support B-cell maturation, affinity maturation, and differentiation into plasma cells (8,9). The presence of germinal center B cells and TLS has been associated with favorable prognosis and enhanced immunotherapy responses in multiple cancers, including breast cancer (10).

*CCDC69* is a coiled-coil domain-containing protein that has been sporadically implicated in the regulation of cell division and tumor-associated biological processes, yet its role in breast cancer remains largely unclear. Recent bioinformatic analyses indicate that *CCDC69* is downregulated in breast cancer tissues and that reduced expression (11) is associated with unfavorable clinical outcomes (12). Moreover, immune-related analyses suggest that *CCDC69* expression is positively correlated with the infiltration of antitumor immune cells (13,14), implicating a potential role in the tumor immune microenvironment and immunotherapeutic responsiveness.

In this study, we performed an integrated multi-omics analysis to investigate the role of *CCDC69* in TNBC. We first examined the expression pattern and prognostic value of *CCDC69* using pan-cancer and TNBC transcriptomic datasets. We then characterized the cellular sources of *CCDC69* using single-cell RNA sequencing data and explored its spatial distribution within the tumor microenvironment using spatial transcriptomics. Finally, we evaluated the relationship between *CCDC69* expression, germinal center B-cell infiltration, and patient prognosis. Our findings revealed that *CCDC69* is closely associated with B-cell and germinal center immune niches in TNBC and may serve as a potential biomarker of immune activation and favorable clinical outcomes.

## Material and Methods

### Data collection

Transcriptomic data and corresponding clinical information for breast cancer were obtained from publicly available datasets. TNBC samples were identified based on molecular subtype classification using the PAM50 gene expression signature (15), in which basal-like tumors were considered representative of triple-negative breast cancer. Single-cell RNA sequencing (scRNA-seq) datasets were obtained from the Gene Expression Omnibus (GEO), including the TNBC tumor microenvironment dataset GSE263995 and the immune-cell-specific dataset GSE169246. Spatial transcriptomic data from breast cancer tissues were obtained from the publicly available dataset GSE210616.

### Bulk transcriptomic analyses

Differential expression analysis was performed to evaluate the expression level of *CCDC69* between tumor and normal tissues across cancer types. For TNBC samples, patients were stratified into *CCDC69* high-expression and low-expression groups according to the median expression level.

To investigate the relationship between *CCDC69* expression and the tumor immune microenvironment, immune cell infiltration levels were estimated using CIBERSORT (16) and single-sample gene set enrichment analysis (ssGSEA). CIBERSORT was applied to estimate the relative proportions of 22 immune cell types using the LM22 signature matrix, while ssGSEA was used to calculate enrichment scores for immune-related signatures. Spearman correlation analysis was performed to evaluate associations between *CCDC69* expression and immune infiltration levels.

The association between somatic copy number alterations (SCNA) of *CCDC69* and immune cell infiltration was also evaluated. SCNA categories included deep deletion, arm-level deletion, diploid/normal, arm-level gain, and high amplification, and immune infiltration levels were compared across these genomic alteration groups.

### Single-cell transcriptomic analyses

Single-cell RNA sequencing data from GSE263995 were analyzed using the Seurat R package (17). Quality control filtering was applied to remove low-quality cells based on the number of detected genes, total counts, and mitochondrial gene expression levels. After normalization and scaling, principal component analysis (PCA) was performed for dimensionality reduction. To correct for potential batch effects across samples, the Harmony algorithm (18) was applied for batch correction. Cell clusters were then identified using a graph-based clustering algorithm and visualized using Uniform Manifold Approximation and Projection (UMAP). Cell-type annotation was performed based on canonical marker genes, and feature plots were generated to examine the distribution of *CCDC69* expression across cell populations.

To further characterize immune cell subtypes associated with *CCDC69*, the immune-cell-specific dataset GSE169246 was analyzed using a similar pipeline. Germinal center B-cell clusters were annotated based on marker genes including *AICDA*, *SERPINA9*, *MEF2B*, and *RGS13*. The correlation between *CCDC69* expression and the germinal center transcription factor BCL6 was evaluated using Spearman correlation analysis.

### Spatial transcriptomic analysis

Spatial transcriptomic data from GSE210616 were analyzed using the Seurat R package. Raw spatial gene expression matrices generated from the 10x Genomics Visium platform (19) were loaded using the Load10X_Spatial function. Multiple spatial samples were integrated by merging individual Seurat objects to generate a combined spatial dataset.

Quality control filtering was performed to remove low-quality spatial spots. Spots with fewer than 200 detected genes, more than 7,000 detected genes, or mitochondrial gene content greater than 20% were excluded. Mitochondrial gene percentages were calculated using the PercentageFeatureSet function. After filtering, gene expression data were normalized using the LogNormalize method with a scale factor of 10,000. Highly variable genes were identified using the FindVariableFeatures function (vst method, top 2,000 genes), followed by data scaling using the ScaleData function. To quantify pathway and immune-related activities across spatial spots, module scores were calculated using the AddModuleScore function. Module scores were generated for *CCDC69*, immune-related signatures, and selected signaling pathways. A germinal center (GC) gene signature, including genes such as *BCL6, AICDA*, *MEF2B*, *RGS13*, and *LMO2*, was used to calculate GC module scores.

Spatial proximity analysis was performed to evaluate the spatial relationship between *CCDC69*-enriched regions and immune-related regions. Spatial coordinates of each spot were extracted using the GetTissueCoordinates function. Spots with high *CCDC69* module scores were defined as those within the top quartile (75th percentile) of the score distribution. Similarly, immune-enriched or GC-enriched spots were defined based on the top quartile of their respective module scores. The nearest-neighbor distances between *CCDC69*-high spots and immune- or GC-high spots were calculated using the k-nearest neighbors algorithm in the FNN R package.

To characterize spatial immune niches within tumor tissues, spatial spots were clustered based on immune-related and pathway module scores using k-means clustering. This analysis classified spatial regions into distinct microenvironmental niches, such as immune-rich and immune-desert areas. Differences in *CCDC69* module scores across spatial niches were evaluated using the Wilcoxon rank-sum test. Finally, the relationship between *CCDC69* expression and GC-related signatures was assessed using Spearman correlation analysis across spatial spots.

### Validation analyses in the TCGA cohort

Tumor purity estimates were obtained using computational approaches. Spearman correlation and partial correlation analyses were conducted to evaluate the relationships among *CCDC69* expression, AICDA expression, and tumor purity.

To further explore the relationship between *CCDC69* expression and germinal center B-cell activity, a GC gene signature was constructed based on established GC-associated genes, including *BCL6*, *AICDA*, *CD38*, *CD19*, *CD79A*, *CD83*, and *MEF2B*. GC module scores and germinal center B-cell (GCB) infiltration scores were calculated using the ssGSEA method. Samples were stratified into GCB-high and GCB-low groups according to the median score. The association between *CCDC69* expression and GC-related signatures was evaluated using Spearman correlation analysis.

Receiver operating characteristic (ROC) curve analysis was performed to evaluate the predictive performance of *CCDC69* expression in distinguishing GCB-high and GCB-low tumors, and the area under the curve (AUC) was calculated.

Logistic regression analysis was performed to evaluate the association between *CCDC69* expression and GCB enrichment. Variables including *CCDC69* expression and clinicopathological characteristics were included in the univariate model, and variables with potential significance were further included in the multivariate logistic regression analysis. Odds ratios (ORs) and 95% confidence intervals (CIs) were calculated.

### Survival analysis

Kaplan-Meier survival analyses were performed using the Kaplan-Meier Plotter database, an online tool integrating multiple breast cancer transcriptomic datasets. Patients were stratified into high- and low-expression groups according to the median expression level of *CCDC69*. Overall survival (OS), recurrence-free survival (RFS), and distant metastasis-free survival (DMFS) were evaluated, and statistical significance was assessed using the log-rank test.

### External validation using the GSE58812 cohort

Gene expression data and corresponding clinical information for the validation cohort were obtained from the GEO database (GSE58812). A total of 107 TNBC samples with available survival data were included. Expression data were normalized according to the original platform annotation, and probes were mapped to gene symbols; when multiple probes corresponded to the same gene, the average expression value was used. Patients were stratified into high- and low-*CCDC69* expression groups based on the median expression level. The GCB infiltration score for each sample was calculated using ssGSEA based on a predefined GCB-related gene signature, and samples were further classified into GCB-high and GCB-low groups using the median score as the cutoff. Differences in GCB infiltration between groups were assessed using the Wilcoxon rank-sum test, and the correlation between *CCDC69* expression and GCB score was evaluated using Spearman's rank correlation coefficient. ROC curve analysis was performed to assess the ability of *CCDC69* expression to distinguish GCB-high from GCB-low tumors, and the AUC was calculated. Kaplan-Meier survival analysis was conducted to evaluate OS and metastasis-free survival (MFS), with statistical significance assessed using the log-rank test.

### Statistical analysis

All statistical analyses were performed using R software (version 4.2.0, Austria). Continuous variables were compared using the Wilcoxon rank-sum test, and correlation analyses were conducted using Spearman correlation coefficients. A two-sided P value <0.05 was considered statistically significant.

## Results

### *CCDC69* expression was associated with prognosis and immune infiltration in TNBC

To investigate the clinical relevance of *CCDC69* in cancer, we first examined its expression across multiple tumor types using data from The Cancer Genome Atlas (TCGA) and GTEx databases. Pan-cancer analysis revealed that *CCDC69* expression was significantly reduced in tumor tissues compared with normal tissues in several cancer types, including breast cancer (Figure 1A).

**Figure 1:**
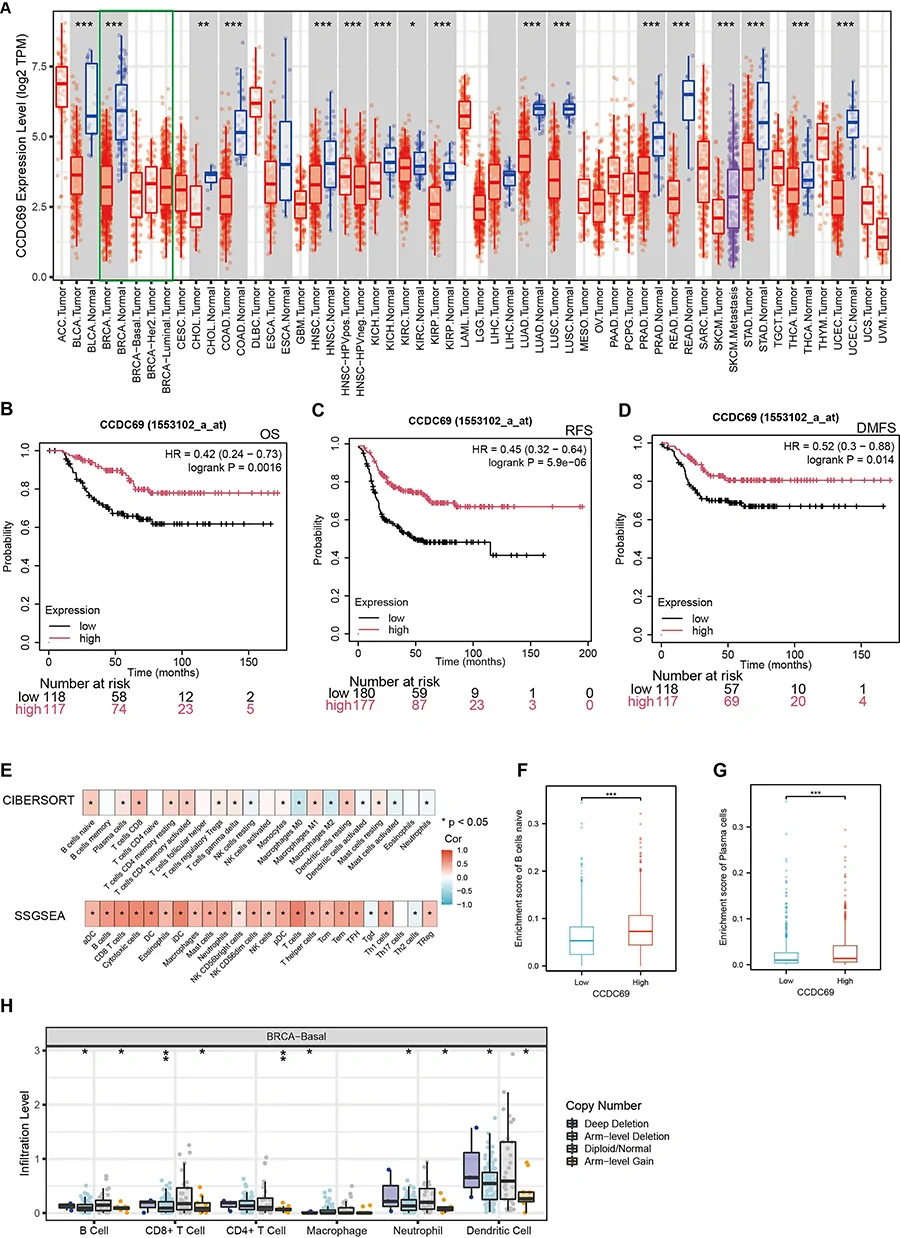
Expression pattern, prognostic value, and immune infiltration of *CCDC69* in triple-negative breast cancer (TNBC). **A**, Pan-cancer expression analysis of *CCDC69*. **B**, Kaplan-Meier analysis of overall survival (OS) in TNBC; **C**, recurrence-free survival (RFS); and **D**, distant metastasis-free survival (DMFS). **E**, Correlation between *CCDC69* expression and immune cell infiltration estimated by CIBERSORT and ssGSEA. Spearman correlation analysis. **F** and **G**, Comparison of B-cell and plasma-cell infiltration scores between *CCDC69* high- and low-expression groups. **H**, Association between somatic copy number alterations (SCNA) of *CCDC69* and immune cell infiltration levels in basal-like breast cancer. B-D: Survival curves were generated using the Kaplan-Meier method and compared using the log-rank test. A and F-H: Data are reported as median and interquartile range. *P<0.05, **P<0.01, ***P<0.001; Wilcoxon rank-sum test.

We next evaluated the prognostic significance of *CCDC69* in triple-negative breast cancer. TNBC patients with high *CCDC69* expression exhibited significantly better overall survival than those with low expression (Figure 1B). Similarly, higher *CCDC69* expression was associated with longer RFS and DMFS (Figure 1C and D).

Given the strong clinical association, we further explored whether *CCDC69* expression is linked to the tumor immune microenvironment. *CCDC69* expression was positively correlated with multiple immune cell populations (Figure 1E). In particular, B-cell and plasma-cell infiltration scores were significantly higher in tumors with high *CCDC69* expression (Figure 1F and G).

In addition, we examined the relationship between SCNA of *CCDC69* and immune infiltration in basal-like breast cancer. Arm-level deletions of *CCDC69* were associated with reduced infiltration of B cells, CD8+ T cells, neutrophils, and dendritic cells, whereas arm-level gains were associated with increased infiltration of these immune populations. Deep deletions were also associated with macrophage infiltration (Figure 1H). These findings indicated a close relationship between *CCDC69* expression and the immune landscape of TNBC.

### Single-cell transcriptomic analysis revealed enrichment of *CCDC69* in B lineage and germinal center B cells

We analyzed the single-cell RNA-sequencing dataset GSE263995. Unsupervised clustering identified multiple cellular populations within the tumor microenvironment (Figure 2A and B). Feature plot analysis showed that *CCDC69* expression was predominantly enriched in clusters 4 and 10 (Figure 2C).

**Figure 2:**
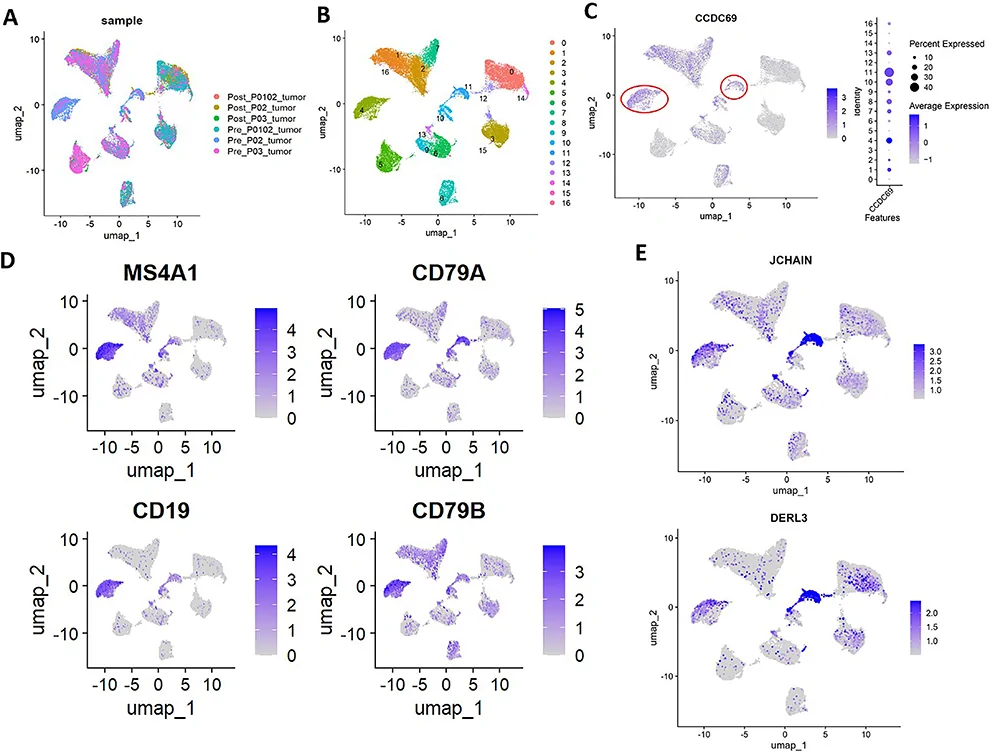
Single-cell transcriptomic analysis identified *CCDC69* enrichment in B lineage cells. **A** and **B**, UMAP visualization and clustering of the TNBC single-cell dataset GSE263995. **C**, Feature plot showing *CCDC69* expression across cell clusters. **D** and **E**, Expression of canonical B-cell markers (*MS4A1, CD79A, CD19, CD79B*) and plasma-cell markers (*JCHAIN, DERL3*).

Cell-type annotation based on canonical markers revealed that cluster 4 expressed B-cell markers (*MS4A1, CD79A, CD19, CD79B*), whereas cluster 10 expressed plasma-cell markers (*JCHAIN* and *DERL3*) (Figure 2D and E), indicating that *CCDC69* expression is primarily localized within B-lineage immune cells.

To further characterize the B-cell subtype associated with *CCDC69* expression, we analyzed an independent immune-cell-specific single-cell dataset (GSE169246). UMAP clustering identified multiple immune populations (Figure 3A and B), among which cluster 16 showed the highest *CCDC69* expression (Figure 3C). This cluster displayed strong expression of germinal center B-cell markers, including *AICDA*, *SERPINA9*, *MEF2B*, and *RGS13* (Figure 3D). Consistently, correlation analysis demonstrated a positive association between *CCDC69* and the germinal center transcription factor *BCL6* (r=0.25, P<0.01; Figure 3E). These results suggest that *CCDC69* expression is preferentially enriched in germinal center B cells within the TNBC immune microenvironment.

**Figure 3:**
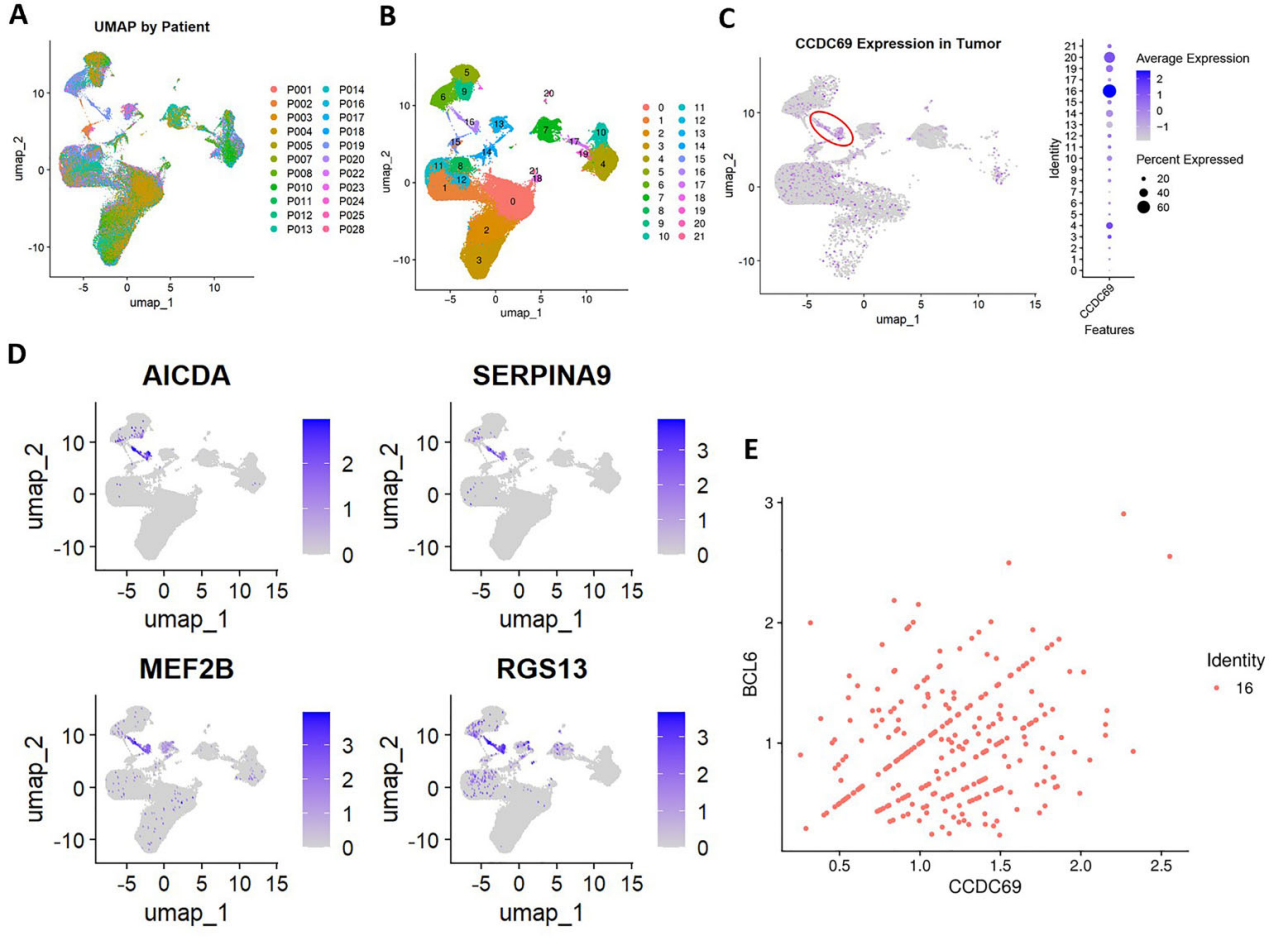
*CCDC69* expression is enriched in germinal center B cells in immune-specific single-cell data. **A** and **B**, Uniform Manifold Approximation and Projection (UMAP) visualization of immune cell populations in dataset GSE169246. **C**, Feature plot showing *CCDC69* expression across immune cell clusters. **D**, Expression of germinal center B-cell markers (*AICDA, SERPINA9, MEF2B, RGS13*). **E**, Correlation analysis between *CCDC69* and BCL6. Spearman correlation analysis.

### Spatial transcriptomics revealed localization of *CCDC69* in B-cell and germinal center niches

To further determine whether *CCDC69* is spatially associated with B-cell immune niches, we analyzed spatial transcriptomic data from breast cancer tissues. After quality control of spatial spots (Figure 4A and B), spatial proximity analysis showed that *CCDC69*-high spots were located closer to B-cell-enriched regions, indicating a spatial co-localization pattern within the tissue (Figure 4C and D).

**Figure 4:**
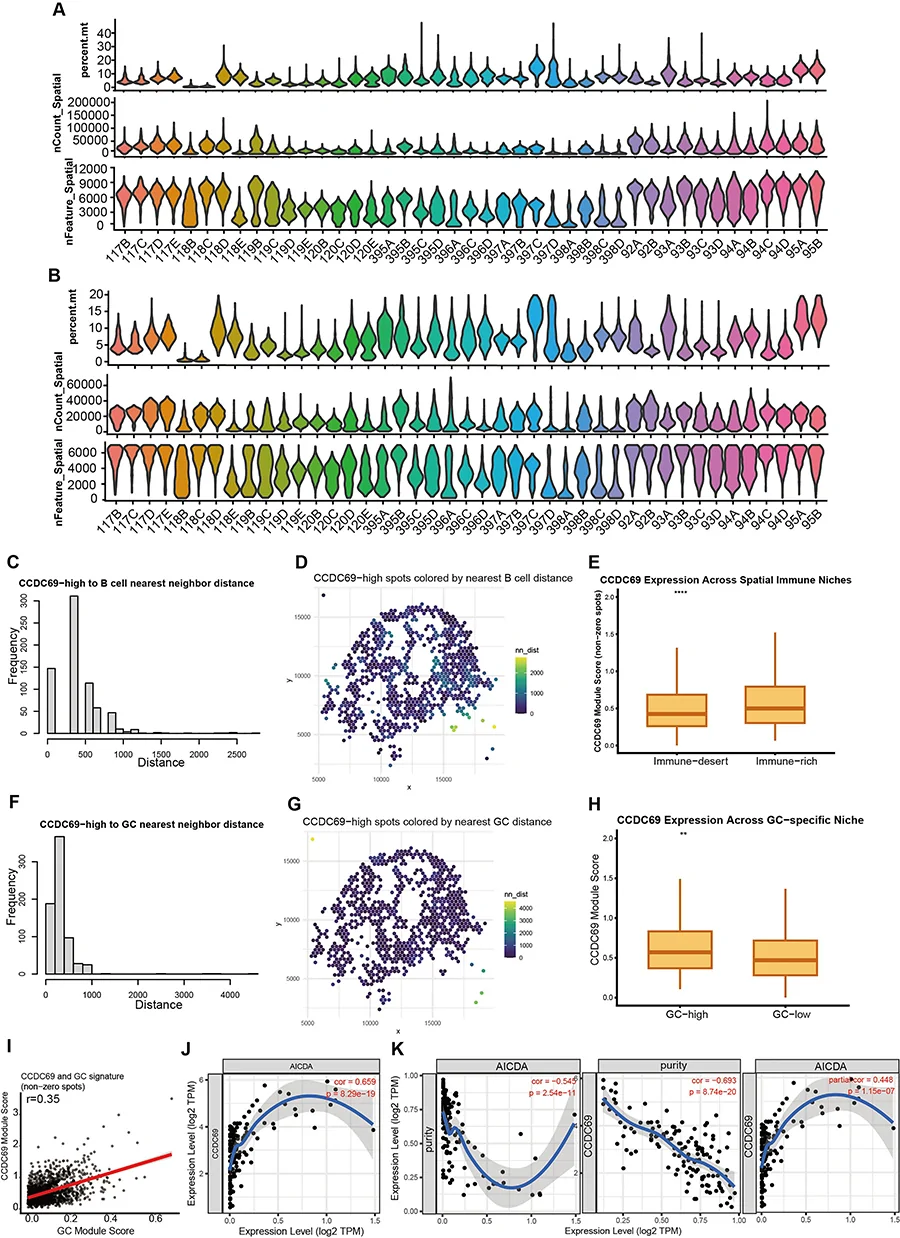
Spatial association of *CCDC69* with B-cell and germinal center (GC) niches. **A** and **B**, Quality control metrics of spatial transcriptomic spots. **C**, Nearest-neighbor distance analysis between *CCDC69*-high spots and B-cell-enriched regions. **D**, Spatial co-localization patterns of *CCDC69* and B-cell signatures. **E**, Comparison of *CCDC69* expression between immune-rich and immune-desert niches. **F** and **G**, Spatial proximity and distribution of *CCDC69-*high spots and GC-high regions. **H**, Comparison of *CCDC69* expression in GC-rich and GC-poor areas. **I**, Correlation between *CCDC69* expression and GC module score. **J**, Correlation between *CCDC69* and *AICDA* expression in the triple-negative breast cancer cohort. **K**, Partial correlation analysis among *CCDC69*, *AICDA*, and tumor purity. Data are reported as median and interquartile range. **P<0.01, ****P<0.0001; Wilcoxon rank-sum test. Correlations were assessed using Spearman correlation analysis.

Based on immune scores, spatial spots were further categorized into immune-rich and immune-desert niches. *CCDC69* expression was significantly higher in immune-rich regions (Figure 4E), supporting its association with immune-active microenvironments.

Given the enrichment of *CCDC69* in GCB at the single-cell level, we next examined its spatial relationship with GC niches. Using a GC gene signature, spatial proximity analysis showed that *CCDC69*-high spots were located closer to GC-high regions (Figure 4F), and spatial mapping further demonstrated a clear neighboring distribution between these regions (Figure 4G). In addition, *CCDC69* expression was significantly elevated in GC-rich areas (Figure 4H). Spearman correlation analysis further revealed a positive correlation between *CCDC69* expression and GC module scores (r=0.35, P<0.01; Figure 4I).

To validate these findings in an independent dataset, we analyzed the TCGA TNBC cohort. *CCDC69* expression showed a strong positive correlation with *AICDA*, a representative marker of germinal center B cells (r=0.659; Figure 4J). Because tumor purity may influence immune-related gene expression, we further examined the relationships among *CCDC69*, *AICDA*, and tumor purity. Tumor purity was negatively correlated with both *AICDA* and *CCDC69*, and the positive correlation between *CCDC69* and *AICDA* remained significant after adjusting for tumor purity (Figure 4K).

### *CCDC69* was strongly associated with GCB infiltration and favorable prognosis in TNBC

Patients in the TCGA TNBC cohort were divided into high- and low-*CCDC69* expression groups according to the median expression level. The clinicopathological characteristics of the two groups are summarized in Table 1. No significant differences were observed in age, tumor stage, T stage, or N stage between the two groups.

**Table 1:** Clinicopathological characteristics of patients in the TCGA triple-negative breast cancer (TNBC) cohort stratified by *CCDC69* expression level.

Variable	High (n=110)	Low (n=111)	P value
Age, years (mean±SD)	55.05±12.52	54.88±11.08	0.919
Tumor stage, n (%)			0.245
I-II	92 (83.6)	92 (82.9)	
III-IV	15 (13.6)	22 (19.8)	
Unknown	3 (2.7)	1 (0.9)	
T stage, n (%)			0.462
T1	29 (26.4)	16 (14.4)	
T2	66 (60.0)	78 (70.3)	
T3-T4	15 (13.6)	16 (14.4)	
Unknown	0 (0.0)	1 (0.9)	
N stage, n (%)			0.358
N0	64 (58.2)	68 (61.3)	
N1	35 (31.8)	27 (24.3)	
N2-N3	10 (9.1)	15 (13.5)	
Unknown	1 (0.9)	0 (0.0)	

Data are reported as means and SD or number and percentage. Student’s *t*-test for continuous variables and chi-squared test for categorical variables.

To further quantify the association between *CCDC69* expression and GCB infiltration, we analyzed the TCGA TNBC cohort. The GCB infiltration score was significantly higher in the *CCDC69* high-expression group compared with the low-expression group (Figure 5A). Consistently, *CCDC69* expression showed a strong positive correlation with the GCB score (r=0.79; Figure 5B).

**Figure 5:**
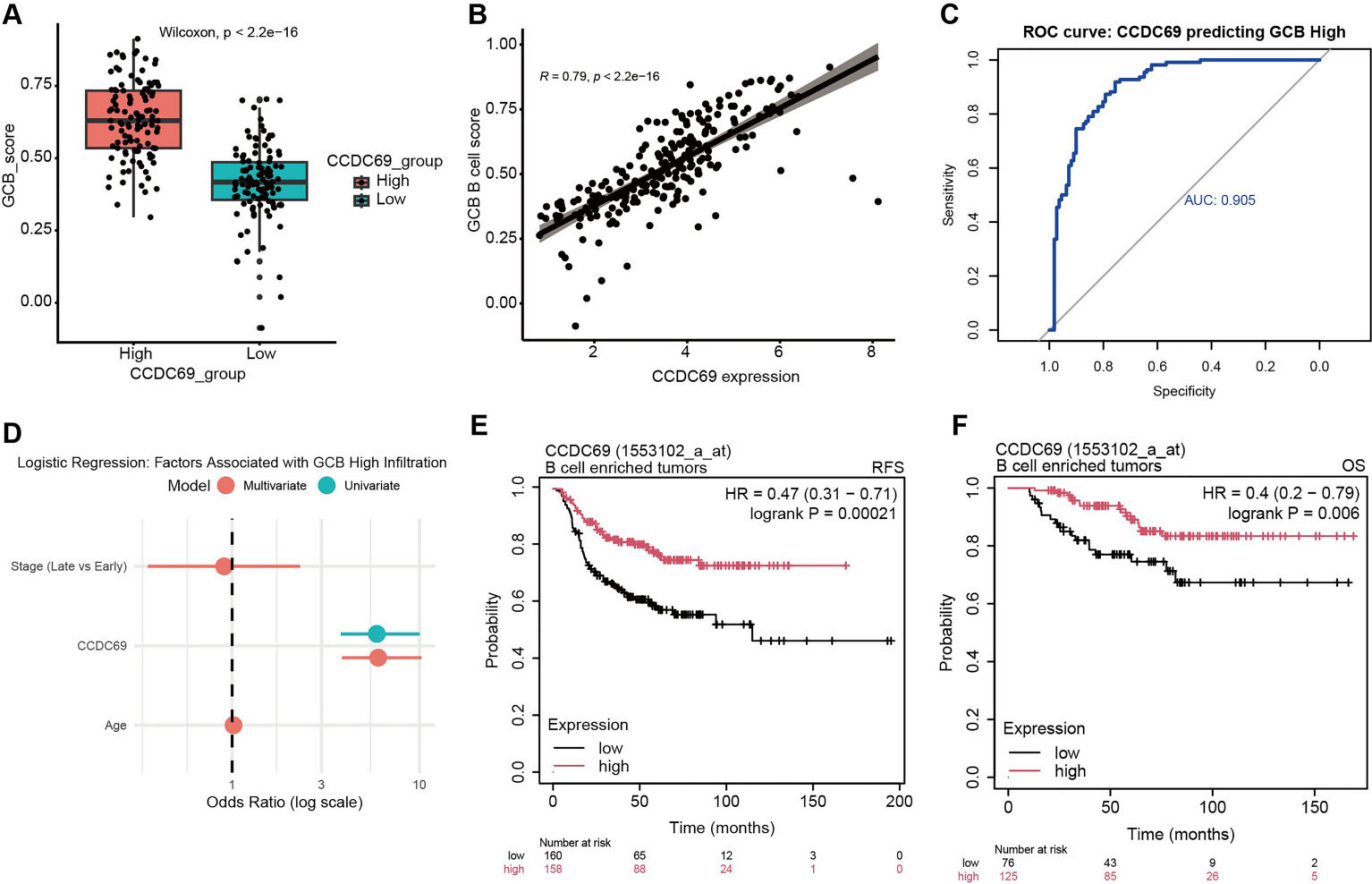
Association of *CCDC69* expression with germinal center B-cell (GCB) infiltration and prognosis in TNBC (triple-negative breast cancer). **A**, Comparison of GCB infiltration scores between *CCDC69* high- and low-expression groups; Wilcoxon rank-sum test. **B**, Correlation between *CCDC69* expression and GCB score. Spearman correlation analysis. **C**, ROC analysis evaluating the predictive performance of *CCDC69* for GCB enrichment. **D**, Logistic regression analysis of *CCDC69* and GCB infiltration. **E** and **F**, Kaplan-Meier analyses of recurrence-free survival (RFS) and overall survival (OS) in B-cell-enriched TNBC samples stratified by *CCDC69* expression.

ROC analysis demonstrated that *CCDC69* expression effectively discriminated GCB-high from GCB-low tumors, with an AUC of 0.905 (Figure 5C). Logistic regression analysis further confirmed that *CCDC69* expression was significantly associated with GCB enrichment in both univariate and multivariate models (Figure 5D; Supplementary Tables S1 and S2).

Finally, to evaluate the clinical relevance of this association, survival analyses were performed in B-cell-enriched TNBC samples from the Kaplan-Meier Plotter database. Patients with high *CCDC69* expression exhibited significantly longer RFS and OS than those with low expression (Figure 5E and F).

### External validation confirmed the association between *CCDC69* expression and GCB infiltration in TNBC

To validate our findings, we analyzed an independent TNBC cohort from GSE58812 (n=107). Consistent with the TCGA results, the GCB infiltration score was significantly higher in the *CCDC69* high-expression group (Figure 6A). Moreover, *CCDC69* expression remained strongly positively correlated with GCB infiltration (r=0.71; Figure 6B). ROC analysis further confirmed that *CCDC69* expression could effectively discriminate GCB-high from GCB-low tumors (AUC=0.833; Figure 6C). Due to the limited sample size of the validation cohort, survival analyses did not reach statistical significance; however, a consistent trend toward improved OS and MFS was observed in patients with higher *CCDC69* expression and GCB enrichment (Figure 6D and E).

**Figure 6:**
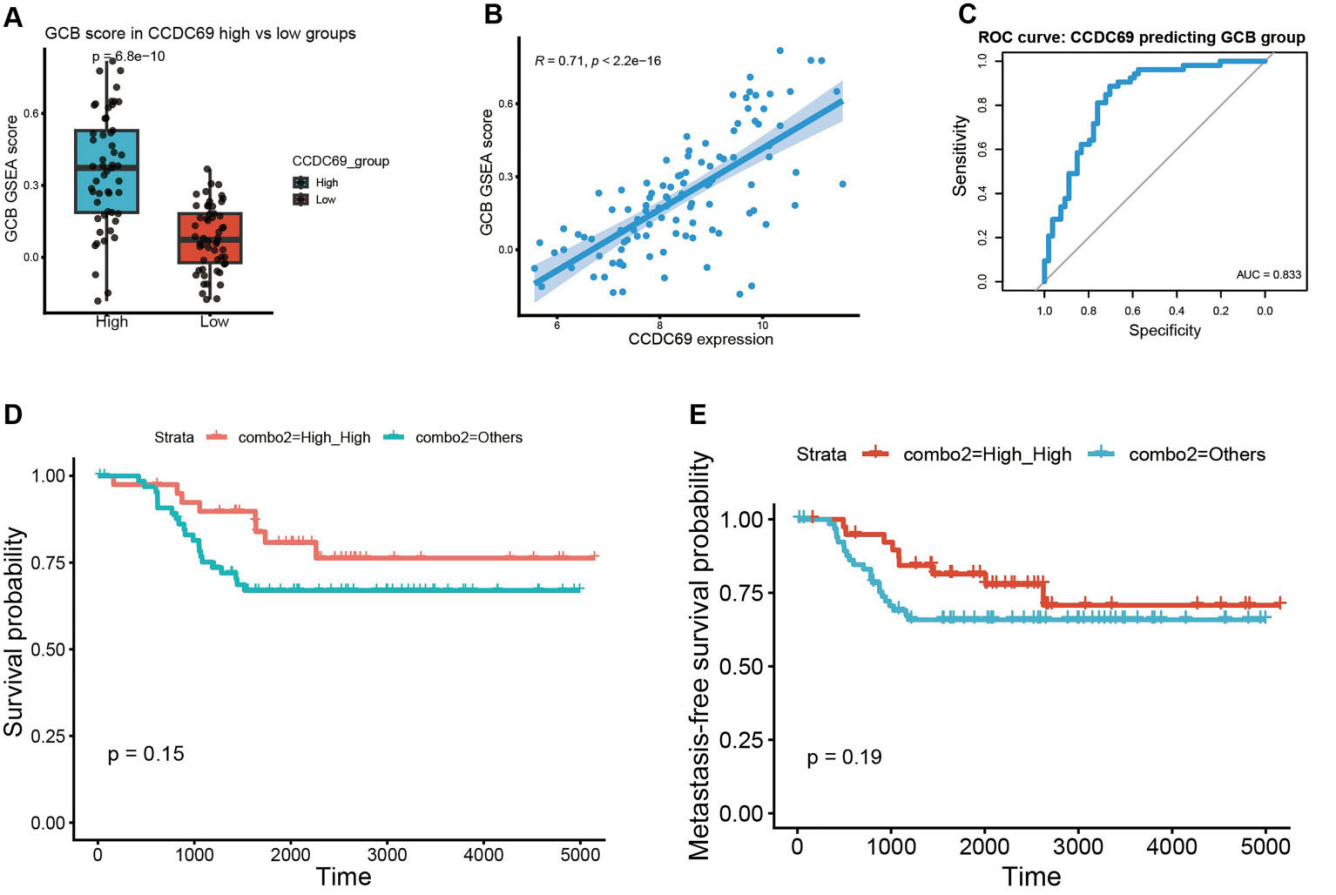
External validation of the association between *CCDC69* expression and germinal center B-cell (GCB) infiltration in the GSE58812 triple-negative breast cancer (TNBC) cohort. **A**, Comparison of GCB infiltration scores between *CCDC69* high- and low-expression groups. Data are reported as median and interquartile range. Wilcoxon rank-sum test. **B**, Correlation between *CCDC69* expression and GCB-related GSEA score. Spearman correlation analysis. **C**, ROC curve showing the performance of *CCDC69* expression in distinguishing GCB-high and GCB-low tumors. **D**, Kaplan-Meier curve of overall survival (OS) stratified by *CCDC69* expression and GCB enrichment. **E**, Kaplan-Meier curve of metastasis-free survival (MFS) stratified by *CCDC69* expression and GCB enrichment.

## Discussion

In this study, we performed an integrative multi-omics analysis to investigate the role of *CCDC69* in the tumor immune microenvironment of TNBC. Our results demonstrated that *CCDC69* expression was significantly associated with favorable clinical outcomes and increased immune infiltration in TNBC. Through single-cell transcriptomic analysis, we further identified that *CCDC69* expression was predominantly enriched in B lineage immune cells, particularly GCB. Spatial transcriptomic analysis revealed that *CCDC69*-high regions spatially co-localize with B-cell-enriched and germinal center niches within tumor tissues. Finally, quantitative analyses in the TCGA TNBC cohort showed that *CCDC69* expression was strongly correlated with GCB infiltration and can effectively distinguish GCB-enriched tumors. Collectively, these findings suggest that *CCDC69* represents a potential marker of B-cell-driven immune activation and favorable prognosis in TNBC.

Accumulating evidence has highlighted the critical role of B cells in shaping antitumor immunity (20- [Bibr B21] 22). Beyond their classical role in antibody production, tumor-infiltrating B cells can function as antigen-presenting cells and secrete cytokines that regulate both innate and adaptive immune responses (23). In many tumors, B cells are spatially organized within TLS, where they frequently undergo germinal center-like reactions that facilitate affinity maturation and differentiation into antibody-secreting plasma cells (23). The formation of TLS and the presence of germinal center B cells have been increasingly recognized as hallmarks of immunologically active tumor microenvironments and have been associated with improved clinical outcomes across multiple cancer types (24). Moreover, recent studies have demonstrated that TLS-rich tumors exhibit enhanced responses to immune checkpoint blockade (25), suggesting that B-cell-mediated humoral immunity may play an important role in antitumor immune surveillance and therapeutic responsiveness. Consistent with these observations, our analyses revealed that *CCDC69* expression was strongly correlated with B-cell and plasma-cell infiltration. These findings suggest that elevated *CCDC69* expression may reflect the presence of TLS-like immune niches and germinal center-associated B-cell activity within the tumor microenvironment, thereby indicating a more immune-active tumor state characterized by enhanced humoral immune responses.

A key finding of our study is the preferential enrichment of *CCDC69* in germinal center B cells. Single-cell RNA sequencing analysis revealed that *CCDC69* expression is primarily localized to B-cell and plasma-cell populations, with particularly high expression in germinal center B-cell clusters characterized by canonical markers such as *AICDA*, *SERPINA9*, and *MEF2B*. Germinal centers represent specialized microanatomical structures where B cells undergo somatic hypermutation and clonal selection, ultimately generating high-affinity antibody responses (26). The positive correlation between *CCDC69* and the germinal center transcription factor *BCL6* further supports the association between *CCDC69* expression and germinal center biology. These findings suggest that *CCDC69* may be involved in germinal center-associated immune programs or may serve as a transcriptional marker of B cells undergoing germinal center differentiation.

Importantly, spatial transcriptomic analysis provided additional evidence supporting the association between *CCDC69* and germinal center immune niches. We observed that *CCDC69*-high regions were spatially closer to B-cell-enriched areas and germinal center-like regions within tumor tissues. Moreover, *CCDC69* expression was significantly elevated in immune-rich spatial niches and positively correlated with germinal center gene signatures. These spatial observations reinforce the notion that *CCDC69* expression is closely linked to local immune organization within the tumor microenvironment. Given that germinal center reactions within TLS are critical for sustaining antitumor immune responses (27,28), the spatial association between *CCDC69* and germinal center niches may partly explain its strong prognostic significance in TNBC.

Another important observation was the strong quantitative relationship between *CCDC69* expression and GCB infiltration. In the TCGA TNBC cohort, *CCDC69* expression showed a robust correlation with the GCB score and demonstrated high diagnostic accuracy for identifying GCB-enriched tumors. Furthermore, higher *CCDC69* expression was associated with significantly improved recurrence-free survival and overall survival in B-cell-enriched TNBC samples. These findings suggest that *CCDC69* may serve as a surrogate biomarker for germinal center-associated immune activity and could potentially help identify patients with immune-active tumors and better clinical outcomes.

We identified a strong and consistent association between *CCDC69* expression and GCB infiltration in TNBC across independent cohorts. Notably, this relationship was robustly reproduced in the external GSE58812 dataset, where *CCDC69* expression remained significantly correlated with GCB-related signatures and effectively discriminated GCB-high from GCB-low tumors. These findings suggest that *CCDC69* may serve as a reliable surrogate marker of GCB infiltration in TNBC. From a biological perspective, GCB infiltration has been increasingly recognized as an indicator of active humoral immune responses within the tumor microenvironment, which may contribute to improved anti-tumor immunity and favorable clinical outcomes. The strong correlation between *CCDC69* expression and GCB signatures observed in our study implies a potential role of *CCDC69* in shaping or reflecting B-cell-mediated immune responses in TNBC. Although survival analyses in the validation cohort did not reach statistical significance, a consistent trend toward improved overall survival and metastasis-free survival was observed in patients with higher *CCDC69* expression and enhanced GCB infiltration. This may be attributed to the relatively limited sample size of the external cohort, which reduces statistical power. Larger, well-annotated cohorts are warranted to further validate the prognostic value of *CCDC69*.

Several limitations of this study should also be acknowledged. First, our findings are primarily based on computational analyses of publicly available datasets, and experimental validation will be necessary to confirm the functional role of *CCDC69* in B cells and germinal center reactions. Second, although spatial transcriptomic analysis suggests a close association between *CCDC69* and germinal center niches, the spatial resolution of current datasets does not fully capture the cellular interactions within TLS structures. Future studies using high-resolution spatial imaging and functional assays may provide deeper insights into the role of *CCDC69* in TLS formation and immune regulation. In addition, prospective clinical studies will be required to determine whether *CCDC69* expression can predict responses to immunotherapy in TNBC patients.

In summary, our study provided a comprehensive multi-omics characterization of *CCDC69* in TNBC and revealed a strong association between *CCDC69* expression and GCB-mediated immune responses. These findings highlight *CCDC69* as a potential biomarker of immune-active tumor microenvironments and favorable prognosis in TNBC. Further mechanistic investigations may elucidate whether *CCDC69* plays a functional role in B-cell immunity and whether it could serve as a target for enhancing antitumor immune responses.

## Data Availability

All datasets used in this study are publicly accessible and no new datasets were generated.

## Supplementary Materials

Click to view [pdf].
