# Supplementary material for: Integrative multi-omics analysis identifies CCDC69 as a marker associated with germinal center B-cell infiltration in TNBC: development and validation
Source: Braz J Med Biol Res. 2026 Oct 12;59:e15616. doi: 10.1590/1414-431X2026e15616 (PMC13660869; doi:10.1590/1414-431X2026e15616)
Supplement: Supplementary file 1 [file 1414-431X-bjmbr-59-e15616-suppl.pdf]

Table S1. Univariate analysis of the association between *CCDC69* expression and germinal center B-cell enrichment.

Variable	OR	95% CI	P value
CCDC69	5.92	[4.80, 10.04]	<0.001

Table S2. Multivariate analysis of the association between *CCDC69* expression and germinal center B-cell enrichment.

Variable	OR	95% CI	P value
CCDC69	6.44	[4.00, 1.39]	<0.001
Age	1.02	[0.99, 1.06]	0.207
Stage I	2.13	[0.16, 57.90]	0.592
Stage IA	6.32	[0.51, 167.20]	0.181
Stage IIA	4.54	[0.51, 97.80]	0.213
Stage IIB	3.94	[0.42, 87.40]	0.268
Stage IIIA	6.24	[0.54, 155.70]	0.17
Stage IIIB	15.83	[0.58, 919.60]	0.125
Stage IIIC	3.89	[0.10, 143.40]	0.419
Stage IV	0.27	[0.00, 21.50]	0.543
